# A social identity approach to crisis leadership

**DOI:** 10.1111/bjso.12805

**Published:** 2024-09-30

**Authors:** Ilka H. Gleibs

**Affiliations:** ^1^ Department of Psychological and Behavioural Science London School of Economics and Political Science London UK

**Keywords:** crisis, crisis leadership, identity leadership, social identity

## Abstract

This paper discusses the importance of a social identity approach to crisis leadership in the context of global crises such as the Covid‐19 pandemic and emphasizes the interconnected relationships between leaders and followers. I highlight the role of leaders in fostering unity and shaping citizens' responses especially during crises. I discuss the nature of crises and the significant role of political leaders in guiding societal responses and suggest that crisis leadership extends beyond individual competencies and behaviours and involves a shift from individual to collective responses. With this, I introduce the social identity approach to leadership that views leadership as a social influence process and emphasizes the importance of creating a sense of ‘we‐ness’ among followers. Following from that, crisis leadership involves leaders constructing defining features of collective identity and efficacy to address crises appropriately. However, the value of this approach depends on the careful definition of shared identity boundaries, consideration of diverse experiences within society, the evolving nature of crisis leadership over time and potential consequences of crisis leadership. The sustainability of identity leadership, the dynamics of intergroup and subgroup processes, and the complexities of various crises are identified as areas requiring further research.

The current global landscape is marked by the recent Covid‐19 pandemic, ongoing wars across the world, the cost‐of‐living crisis and the looming threats of climate change. Amid these ongoing challenges, the role of leadership in navigating crises has become increasingly critical. In particular, the pandemic highlighted the importance of collective responses in crisis leadership (Haslam et al., 2021; Sriharan et al., 2022; Samad et al., 2022). Understanding how collective responses to crisis influence cooperative action is therefore crucial when we want to understand crisis leadership.

To better understand the psychological dynamics at play during crises, I highlight the significance of the social identity approach to crisis leadership. By focusing on group processes, this approach allows leaders to foster unity and collective efficacy. Building on this, I propose a framework based on social influence and identity processes, which illuminates the interconnected relationships between leaders and followers.

## CONTEXT

A crisis involves difficulty and potential danger, threatening the core functions of groups, organizations or nations. Wu et al. (2021) have defined it as ‘events that are perceived as […] unexpected, highly salient and potentially disruptive’ (p.2; see Riggio & Newstead, 2023 for a history of the definition). Thus, crises are characterized by unpredictability, leaving organizations or nations and their leaders with minimal experience and readiness to handle them. Crises can be relatively sudden, such as a pandemic, or longer lasting, like the climate emergency. While the latter may not be unexpected, its impacts are often unpredictable and widespread, affecting various aspects of society and the environment. Regardless, crises demand *collective* response and often necessitate significant changes in policies and behaviours.

In such turbulent times, leadership plays a pivotal role in guiding societal responses, as (political) leaders influence not only policies but also how citizens process information, interact and manage emotional distress (Geiger et al., 2021; Homer‐Dixon et al., 2021; Leung et al., 2022) and shape societal responses. Therefore, their role is crucial in creating a social environment (e.g. on a national level) where citizens are motivated to take actions (e.g. like wearing masks) and feel capable of adapting to crisis challenges (e.g. decreasing infection). Thus, citizens' reactions and effective leadership are closely intertwined. While crisis leadership is frequently framed as a managerial, individualistic task—focused on technical preparedness (Riggio & Newstead, 2023)—I argue that it also poses significant psychological challenges. These challenges require leaders to consider the collective experience of those they lead, rather than focusing solely on individual capabilities. Thus, crisis leadership, like leadership in general (see Haslam et al., 2024), must extend beyond specific management skills or competencies that individual leaders possess or lack and focus on a relational approach, where leaders encourage people to ‘act as if they are a group’ (Weick & Roberts, 1993), which is at the heart of a social identity approach to leadership (Haslam et al., 2020).

## THEORETICAL BACKGROUND

Crisis leadership is defined as ‘the influencing process that occurs between leader(s) and stakeholder in the context of […] crisis, as opposed to run‐of‐the‐mill […] challenges over the various stages of the crisis lifecycle’. (Collins et al., 2023, p.2). It has garnered interest across political science, health care, public health and organizational psychology and beyond, particularly since the Covid‐19 pandemic (Sriharan et al., 2022; Haslam et al., 2021). However, recent reviews highlight that the field lacks a clear theoretical framework addressing crisis leadership (Collins et al., 2023; Riggio & Newstead, 2023; Wu et al., 2021). Although Riggio and Newstead (2023) mention several theories that explicitly deal with crisis,^1^ like many other models in leadership research, they rely heavily on the role of individual leaders and their specific qualities, competencies and behaviours rather than focusing on the social processes that are at play during crisis (Haslam et al., 2024). Thus, the focus on collective processes is largely absent from contemporary crisis leadership research and practice and represents a mismatch between the leadership psychology presented in those approaches and the requirements of leadership in crisis.

Therefore, I argue that people's reaction to societal crisis and how this is shaped by leadership should be understood through the lens of the Social Identity Approach to Leadership (Haslam et al., 2020; Hogg, 2001; Reicher et al., 2005). This approach is based on social identity (Tajfel et al., 1979) and self‐categorization theory (Turner et al., 1987), which highlight the importance of internalized group memberships for our thinking, feeling and behaviour. Thus, a social identity is the sense of self associated with a knowledge that one belongs to a particular social group (e.g. a nation, a community, an organization), and that this group membership is important and meaningful (Tajfel, 1974). Further, the social identity approach of leadership sees social identities (e.g. people's sense of who they are based on the groups they belong to) as the key for social influence processes between leaders and followers. Leadership is then defined as a social influence process through which someone alters and shapes the thinking and doing of others and has the capacity to shape people's perception of their social reality and is inherently related to the groups we belong to (Reicher & Hopkins, 1996, 2000). In addition, leadership is also interwoven with followership as ‘there is no leadership if no‐one follows’ (Platow et al., 2015, p.20) and shapes collective mobilization (Klein & Licata, 2003). Importantly, according to the identity leadership model (Haslam et al., 2020), leaders are effective to the extent that they create a sense of ‘we’ among their followers. They can do so by representing the group (‘Being one of us’), advancing the group's interests (‘Doing it for us’), act as the entrepreneurs of the group's identity and its norms, values and ideals (‘Crafting a sense of us’), and create structures and activities that give importance to the group (‘Making us matter’ Haslam et al., 2020; Steffens et al., 2014). Crucially, in this conceptualization of leadership, power is not held by a specific individual but distributed and shared among the members of the group. Thus, leaders' power comes from support, cooperation and active participation of the group and the approach shifts from a traditional view of leadership as ‘power over others’ (Haslam et al., 2020, p. 57) to a collective responsibility.

A societal crisis, like a pandemic, might be a situation that triggers collective threat, which increases the perception that society is vulnerable and might therefore activate a psychological need to restore safety (Contu et al., 2024). For example, during the early stages of the Covid‐19 pandemic, Haslam (2020) argued that people deal with the uncertainty and fear of the situation by turning to leaders for guidance. Indeed, a perceived collective threat might amplify the need for a collective response (Ionescu et al., 2024). Following from this, as a crisis evolves, leaders construct defining features of this collective identity to address the crisis effectively and to match its requirements (Haslam & Ellemers, 2011). Thus, it is the shared social identity that leaders must establish which provides the psychological scaffold for a collective response to the crisis (Haslam, 2020). This process of ‘co‐creating’ identity content lies at the core of identity leadership and effective management of the collective ‘we’ becomes crucial (Gleibs et al., 2018). As an example, during a crisis, leaders often shift their rhetoric from ‘I and me’ to ‘we, our and us’. For instance, during the World Trade attacks in 2001, New York City's mayor, Rudy Giuliani, increased his use of ‘we, us, our’ in comparison to first‐person singular (I, me) pronouns (Pennebaker & Lay, 2002, see also Bligh et al., 2004a, 2004b; Montiel et al., 2021) leading to the hypothesis that a crisis prompts leaders to emphasize collective identity (‘we’), which can help them to manage collective behaviours accordingly (e.g. encouraging staying home to protect others).

**FIGURE 1:**
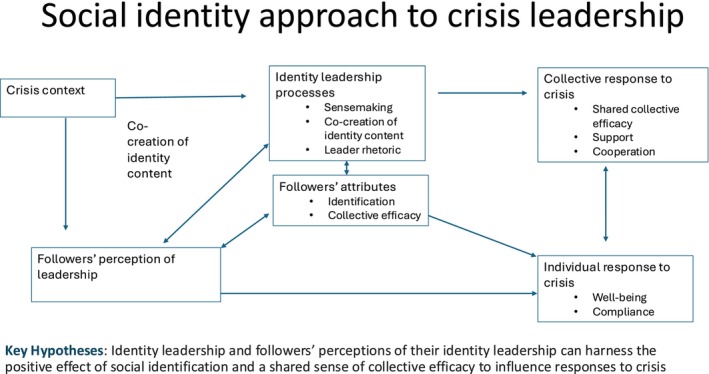
Social identity approach to crisis leadership.

These examples illustrate how leaders can use collective identity to guide behaviour during crises. While such identity‐based leadership has been well‐researched in non‐crisis settings (e.g. see meta‐analysis by Steffens et al., 2021) and cross‐cultural studies (Bracht et al., 2023; Monzani et al., 2024; van Dick et al., 2018), its application to crisis leadership remains less explored. However, initial studies during the Covid‐19 crisis suggest that this lens is valuable for understanding crisis leadership. For instance, Frenzel et al. (2022) argue that national leaders should create an environment where citizens willingly follow specific rules to prevent disease spread. Thus, identity leadership aims to foster a shared sense of identity among citizens, tied to common goals and norms. In a cross‐cultural sample from China, the United States, Germany and Israel, citizens' perception of identity leadership positively correlated with health‐protective behaviours, largely influenced by national identification. Similarly, Krug et al. (2021) found in a work context during the Covid‐19 pandemic that identity leadership within an organization positively impacted social identity continuity, which in turn boosted job satisfaction. Furthermore, Gleibs et al. (2024) examined the relationship between identity leadership, national identification and collective efficacy on mental health during the initial phase of the Covid‐19. They found that the perception that national government acted in accordance with the group (e.g. identity leadership) decreased citizens' psychological ill‐health (e.g. anxiety, depression and stress) and that the effect was driven by national identification but also collective efficacy (hence, the perception that we can do it). These relationships remained mostly stable over time and across different countries. Taken together, these insights highlight the importance of identity leadership in crisis contexts and Figure 1 summarized the theoretical framework of the social identity approach to crisis leadership.

## FURTHER IMPLICATIONS

The theoretical framework and the empirical examples demonstrate that identity leadership promotes collective crisis responses by reinforcing the perception that ‘we (e.g. as a nation) are in this together and can act’. Identity leadership that fosters identity and efficacy becomes a powerful tool for governments (or other institutions of leadership) to shape follower responses during crises. Lessons from the Covid‐19 pandemic highlight that identity leadership revolves around shared social identity, recognizing groups as solutions, unlocking capacity, and defining the group (Haslam et al., 2021). Thus, when leaders promote a shared sense of identity, they are perceived as effective, enhancing identification among group members (e.g. followers) and it is this sense of belongingness and connectedness becomes a resource during challenges (Gleibs et al., 2011; Jetten et al., 2012). Thus, particularly in times of crisis, leaders must collaborate with the groups they lead (hence their ‘followers’) emphasizing a collective ‘we’ (Bruner et al., 2022). Additionally, for collective efficacy (‘we can do it’), a precise definition of the collective (e.g. ‘we’ as a nation, city or organization) that aligns actions (e.g. reducing carbon footprint) with aims (e.g. preventing global warming) is needed (Hamann et al., 2024). This aligning identity content with behaviours is key in crisis leadership. In sum, the presented social identity perspective of crisis leadership transcends beyond specific attributes or individual virtues (Collins et al., 2023; Samad et al., 2022; Sriharan et al., 2022; Wilson & Newstead, 2022) and instead focuses on leadership as a group process that facilitates collective responses.

While the benefits of identity leadership in crisis contexts are clear, several important considerations must be addressed. First, the boundaries of shared identity require careful definition. Haslam et al. (2021) advocate for inclusive ingroups, avoiding fault lines. However, the group's sphere of influence and inclusivity depend on the type of crisis (like a local flood, global pandemic or distant war that impacts local markets), how identities align (such as being a community member or citizen), and the required actions and their costs. Exemplarily, during the pandemic, many countries issued a ‘stay at home’ order. Yet, it was easier for people with ample space, the option to work remotely, and strong support networks to follow this order and way more difficult for individuals who were socially vulnerable, had less resources and/or precarious jobs and those you could not do from home (Fletcher et al., 2021). Hence, the metaphorical ‘boat we were in’ was considerably different for diverse groups in society (Muldoon, 2024). It also demonstrated that leaders often overlook vulnerable groups when making decisions, despite aiming for a collective response. Yet, pre‐existing material and psychological realities shape what can be achieved through social processes (Muldoon, 2024; Templeton et al., 2020) and effective identity leadership depends on understanding these nuances and avoiding fallacies that neglect diverse experiences within society. In addition, social identity processes could also inhibit mobilization for collective efforts. Lau et al. (2022) demonstrated that in the United States the ability to unite in crisis and display preventive behaviours (e.g. wearing a mask) was influenced by political affiliation and was determined by whether citizens perceived their political leader at the time (Donald Trump) as effective (or not). Thus, followers of different (sub‐)groups are not always easily united during crisis and the effects of social identity processes may be attenuated or amplified by perception of prevailing inter‐ and intragroup relations (Gleibs & Haslam, 2016).

Second, crisis leadership evolves over time as the critical event unfolds (Williams et al., 2017). Initially, effective leadership may create a shared identity, but if followers perceive leaders as responsible for the crisis or ineffective in solving it, support may wane. For instance, during the UK's first lockdown in Spring 2020, confidence in the government declined significantly after Dominic Cummings, a key advisor to the Prime Minister, breached lockdown rules (Fancourt et al., 2020). Thus, a key concern for future examination is to understand how identity leadership can be sustained over a period or when a crisis is complex and evolving (Grint, 2024) to focus further on the dynamic interactions between leaders' decisions and policies, behaviour and feedback from followers.

Third, crises perceived as collective threats can increase the desire for ‘strong’ leadership.^2^ Ionescu et al. (2024) showed that a perceived threat to a country's international standing (e.g. status threat) heightened followers' desire for strong leaders. Similarly, Contu et al. (2024) found that viewing Covid‐19 as a collective ecological threat activated the need for cognitive closure, strict norms and a strong leader. Lipman‐Blumen (1996) noted that strong leaders often emerge in times of distress to offer radical solutions. In fact, support for authoritarian and populist leaders has risen in many countries, including Western democracies (Selvanathan et al., 2022), as these leaders claim their strength is necessary to handle crises. Thus, crisis leadership might promote ‘strong leadership’, which in turn could destabilize societies (Maskor et al., 2021). Thus, social identity processes in crisis leadership could drive both constructive and destructive outcomes, including transgressive leadership (Davies et al., 2022; Haslam, Reicher, et al., 2023) and future work should explore how crisis leadership can balance fostering unity while avoiding an overreliance on ‘strong leaders’.

To translate these theoretical insights into practice, leaders can adopt several strategies to foster shared identity and collective action. First, they could use inclusive language to foster shared identity and emphasize collective goals (Bachmann & Gleibs, 2024). It is also essential to recognize the diverse needs of different social groups, tailoring responses to ensure inclusivity (Templeton et al., 2020). Leaders should cultivate collective efficacy by highlighting group successes and emphasizing how individual actions contribute to broader goals. Bridging political and social divides is key, promoting shared identities that transcend differences. Further, crisis leadership must adapt over time, maintaining transparency and trust (e.g. DeNisi & Shin, 2005). Inclusive decision making and preventing the misuse of ‘strong leadership’ are critical to sustaining collective engagement. Finally, training leaders in social identity principles can enhance their ability to foster group cohesion and mobilize effective crisis responses (Haslam, Reutas, et al., 2023). These strategies ensure that leadership remains inclusive, adaptable and effective.

## AUTHOR CONTRIBUTIONS

**Ilka H. Gleibs:** Conceptualization; writing – original draft; investigation; writing – review and editing.

## CONFLICT OF INTEREST STATEMENT

The author has not conflict of interest.

## Data Availability

Data sharing is not applicable to this article as no new data were created or analyzed in this study.

## Footnotes

^1^
Crisis management Theory (Perrow, 1999); Cognitive Resource Theory (Fiedler & Garcia, 1987); Charismatic Leadership Theory (Conger & Kanungo, 1988); Complexity Leadership Theory (Uhl‐Bien & Arena, 2017).

^2^
Strong leaders have been defined as those ‘who promise to overcome difficulties faced by society by any means necessary, including forgoing traditional means or promising to shakeup existing political systems’ (Crimston et al., 2022, p. 671).

## References

[bjso12805-bib-0001] Bachmann, R. , & Gleibs, I. H. (2024). Uncovering the secret life of we‐pronouns in the German parliament. Zeitschrift für Psychologie, 232, 208–220.

[bjso12805-bib-0002] Bligh, M. C. , Kohles, J. C. , & Meindl, J. R. (2004a). Charisma under crisis: Presidential leadership, rhetoric, and media responses before and after the September 11th terrorist attacks. The Leadership Quarterly, 15(2), 211–239.

[bjso12805-bib-0003] Bligh, M. C. , Kohles, J. C. , & Meindl, J. R. (2004b). Charting the language of leadership: A methodological investigation of president bush and the crisis of 9/11. Journal of Applied Psychology, 89(3), 562–574. 15161413 10.1037/0021-9010.89.3.562

[bjso12805-bib-0004] Bracht, E. M. , Monzani, L. , Boer, D. , Haslam, S. A. , Kerschreiter, R. , Lemoine, J. , Steffens, N. K. , Akfirat, S. A. , Avanzi, L. , Barghi, B. , & Dumont, K. (2023). Innovation across cultures: Connecting leadership, identification, and creative behavior in organizations. Applied Psychology, 72(1), 348–388.

[bjso12805-bib-0005] Bruner, M. W. , McLaren, C. D. , Mertens, N. , Steffens, N. K. , Boen, F. , McKenzie, L. , Haslam, S. A. , & Fransen, K. (2022). Identity leadership and social identification within sport teams over a season: A social network analysis. Psychology of Sport and Exercise, 59, 102106.

[bjso12805-bib-0006] Collins, M. D. , Dasborough, M. T. , Gregg, H. R. , Xu, C. , Deen, C. M. , He, Y. , & Restubog, S. L. D. (2023). Traversing the storm: An interdisciplinary review of crisis leadership. The Leadership Quarterly, 34(1), 101661.

[bjso12805-bib-0007] Conger, J. A. , & Kanungo, R. N. (1988). Charismatic leadership in organizations. Sage.

[bjso12805-bib-0008] Contu, F. , Ellenberg, M. , Kruglanski, A. W. , Pantaleo, G. , & Pierro, A. (2024). Need for cognitive closure and desire for cultural tightness mediate the effect of concern about ecological threats on the need for strong leadership. Current Psychology, 43, 11458–11469.

[bjso12805-bib-0009] Crimston, C. R. , Selvanathan, H. P. , & Jetten, J. (2022). Moral polarization predicts support for authoritarian and progressive strong leaders via the perceived breakdown of society. Political Psychology, 43(4), 671–691. 10.1111/pops.12787

[bjso12805-bib-0010] Davies, B. , Leicht, C. , & Abrams, D. (2022). Donald Trump and the rationalization of transgressive behavior: The role of group prototypicality and identity advancement. Journal of Applied Social Psychology, 52(7), 481–495.

[bjso12805-bib-0011] DeNisi, A. , & Shin, S. J. (2005). Psychological communication interventions in mergers and acquisitions. In G. K. Stahl & M. E. Mendenhall (Eds.), Mergers and acquisitions: Managing culture and human resources. Stanford University Press.

[bjso12805-bib-0012] Fancourt, D. , Steptoe, A. , & Wright, L. (2020). The cummings effect: Politics, trust, and behaviours during the COVID‐19 pandemic. The Lancet, 396(10249), 464–465. 10.1016/S0140-6736(20)31690-1 PMC7613216 32771083

[bjso12805-bib-0013] Fiedler, F. E. , & Garcia, J. E. (1987). New approaches to effective leadership and organizational performance. Wiley.

[bjso12805-bib-0014] Fletcher, K. M. , Espey, J. , Grossman, M. K. , Sharpe, J. D. , Curriero, F. C. , Wilt, G. E. , Sunshine, G. , Moreland, A. , Howard‐Williams, M. , & Ramos, J. G. (2021). Social vulnerability and county stay‐at‐home behavior during COVID‐19 stay‐at‐home orders, United States, April 7–April 20, 2020. Annals of Epidemiology, 64, 76–82. 34500085 10.1016/j.annepidem.2021.08.020 PMC8523174

[bjso12805-bib-0015] Frenzel, S. B. , Haslam, S. A. , Junker, N. M. , Bolatov, A. , Erkens, V. A. , Häusser, J. A. , Kark, R. , Meyer, I. , Mojzisch, A. , & Monzani, L. (2022). How national leaders keep ‘us’ safe: A longitudinal, four‐nation study exploring the role of identity leadership as a predictor of adherence to COVID‐19 non‐pharmaceutical interventions. BMJ Open, 12(5), e054980. 10.1136/bmjopen-2021-054980 PMC9091491 35537783

[bjso12805-bib-0016] Geiger, N. , Gore, A. , Squire, C. V. , & Attari, S. Z. (2021). Investigating similarities and differences in individual reactions to the COVID‐19 pandemic and the climate crisis. Climatic Change, 167(1), 1. 34248235 10.1007/s10584-021-03143-8 PMC8253462

[bjso12805-bib-0017] Gleibs, I. H. , Haslam, C. , Jones, J. M. , Alexander Haslam, S. , McNeill, J. , & Connolly, H. (2011). No country for old men? The role of a ‘Gentlemen's Club’ in promoting social engagement and psychological well‐being in residential care. Aging & Mental Health, 15(4), 456–466. 21500012 10.1080/13607863.2010.536137

[bjso12805-bib-0018] Gleibs, I. H. , & Haslam, S. A. (2016). Do we want a fighter? The influence of group status and the stability of intergroup relations on leader prototypicality and endorsement. The Leadership Quarterly, 27(4), 557–573.

[bjso12805-bib-0019] Gleibs, I. H. , Hendricks, K. , & Kurz, T. (2018). Identity mediators: Leadership and identity construction in campaign speeches of American presidential candidates' spouses. Political Psychology, 39(4), 939–956.

[bjso12805-bib-0020] Gleibs, I. H. , Muehlemann, N. S. , & Heliot, Y. H. (2024). How harnessing group processes can enhance mental health in time of crisis: A global investigation of identity leadership . Unpublished manuscript.

[bjso12805-bib-0021] Grint, K. (2024). Is leadership the solution to the wicked problem of climate change? Leadership, 20(2), 77–95.

[bjso12805-bib-0022] Hamann, K. R. , Wullenkord, M. C. , Reese, G. , & van Zomeren, M. (2024). Believing that we can change our world for the better: A triple‐a (agent‐action‐aim) framework of self‐efficacy beliefs in the context of collective social and ecological aims. Personality and Social Psychology Review, 28(1), 11–53. 37386819 10.1177/10888683231178056 PMC10851658

[bjso12805-bib-0023] Haslam, S. A. (2020). Leadership. In J. Jetten , S. D. Reicher , A. A. Haslam , & T. Cruwys (Eds.), Together apart: The psychology of COVID‐19 (pp. 25–30). Sage.

[bjso12805-bib-0024] Haslam, S. A. , Alvesson, M. , & Reicher, S. D. (2024). Zombie leadership: Dead ideas that still walk among us. The Leadership Quarterly, 35(3), 101770.

[bjso12805-bib-0025] Haslam, S. A. , & Ellemers, N. (2011). Identity processes in organizations. In S. Schwartz , K. Luyckx , & V. Vignoles (Eds.), Handbook of identity theory and research (pp. 715–744). Springer. 10.1007/978-1-4419-7988-9_30

[bjso12805-bib-0026] Haslam, S. A. , Reicher, S. D. , & Platow, M. J. (2020). The new psychology of leadership: Identity, influence and power. Routledge.

[bjso12805-bib-0027] Haslam, S. A. , Reicher, S. D. , Selvanathan, H. P. , Gaffney, A. M. , Steffens, N. K. , Packer, D. , Van Bavel, J. J. , Ntontis, E. , Neville, F. , Vestergren, S. , Jurstakova, K. , & Platow, M. J. (2023). Examining the role of Donald Trump and his supporters in the 2021 assault on the US capitol: A dual‐agency model of identity leadership and engaged followership. The Leadership Quarterly, 34(2), 101622.

[bjso12805-bib-0028] Haslam, S. A. , Reutas, J. , Bentley, S. V. , McMillan, B. , Lindfield, M. , Luong, M. , Peters, K. , & Steffens, N. K. (2023). Developing engaged and ‘teamful’ leaders: A randomized controlled trial of the 5R identity leadership program. PLoS One, 18(5), e0286263. 37228145 10.1371/journal.pone.0286263 PMC10212178

[bjso12805-bib-0029] Haslam, S. A. , Steffens, N. K. , Reicher, S. D. , & Bentley, S. V. (2021). Identity leadership in a crisis: A 5R framework for learning from responses to COVID‐19. Social Issues and Policy Review, 15(1), 35–83. 33821168 10.1111/sipr.12075 PMC8013601

[bjso12805-bib-0030] Hogg, M. A. (2001). A social identity theory of leadership. Personality and Social Psychology Review, 5(3), 184–200.

[bjso12805-bib-0031] Homer‐Dixon, T. , Renn, O. , Rockstrom, J. , Donges, J. F. , & Janzwood, S. (2021). A call for an international research program on the risk of a global polycrisis . *SSRN 4058592*.

[bjso12805-bib-0032] Ionescu, O. , Mols, F. , Álvarez, B. , Selvanathan, H. P. , Crimston, C. , & Jetten, J. (2024). ‘We're not as great as we used to be’: Perceived national status threat and the desire for strong leaders. Group Processes & Intergroup Relations, 13684302241265236.

[bjso12805-bib-0033] Jetten, J. , Haslam, C. , & Alexander, S. H. (2012). The social cure: Identity, health and well‐being. Psychology Press.

[bjso12805-bib-0034] Klein, O. , & Licata, L. (2003). When group representations serve social change: The speeches of Patrice Lumumba during the Congolese decolonization. British Journal of Social Psychology, 42(4), 571–593. 14715118 10.1348/014466603322595284

[bjso12805-bib-0035] Krug, H. , Haslam, S. A. , Otto, K. , & Steffens, N. K. (2021). Identity leadership, social identity continuity, and well‐being at work during COVID‐19. Frontiers in Psychology, 12, 684475. 34177738 10.3389/fpsyg.2021.684475 PMC8225939

[bjso12805-bib-0036] Lau, V. W. , Tse, D. C. , Bligh, M. C. , Hong, Y. Y. , Kakarika, M. , Chan, H. W. , & Chiu, C. P. (2022). Not ‘my’ crisis: Social identity and followers' crisis responses to COVID‐19. Analyses of Social Issues and Public Policy, 22(2), 506–535. 10.1111/asap.12316 PMC9349868 35942362

[bjso12805-bib-0037] Leung, C. M. , Ho, M. K. , Bharwani, A. A. , Cogo‐Moreira, H. , Wang, Y. , Chow, M. S. , Fan, X. , Galea, S. , Leung, G. M. , & Ni, M. Y. (2022). Mental disorders following COVID‐19 and other epidemics: A systematic review and meta‐analysis. Translational Psychiatry, 12(1), 205. 35581186 10.1038/s41398-022-01946-6 PMC9110635

[bjso12805-bib-0038] Lipman‐Blumen, J. (1996). The connective edge: Leading in an interdependent world. Jossey‐Bass.

[bjso12805-bib-0039] Maskor, M. , Steffens, N. K. , & Haslam, S. A. (2021). The psychology of leadership destabilization: An analysis of the 2016 US presidential debates. Political Psychology, 42(2), 265–289.

[bjso12805-bib-0040] Montiel, C. J. , Uyheng, J. , & Dela Paz, E. (2021). The language of pandemic leaderships: Mapping political rhetoric during the COVID‐19 outbreak. Political Psychology, 42(5), 747–766. 34230725 10.1111/pops.12753 PMC8250800

[bjso12805-bib-0041] Monzani, L. , Bibic, K. , Haslam, S. A. , Kerschreiter, R. , Wilson Lemoine, J. E. , Steffens, N. K. , Akfirat, S. A. , Ballada, C. J. A. , Bazarov, T. , Aruta, J. J. B. R. , & Avanzi, L. (2024). Political leaders' identity leadership and civic citizenship behavior: The mediating role of trust in fellow citizens and the moderating role of economic inequality. Political Psychology.

[bjso12805-bib-0042] Muldoon, O. T. (2024). The social psychology of trauma: Connecting the personal and the political. Cambridge University Press.

[bjso12805-bib-0043] Pennebaker, J. W. , & Lay, T. C. (2002). Language use and personality during crises: Analyses of mayor Rudolph Giuliani's press conferences. Journal of Research in Personality, 36(3), 271–282.

[bjso12805-bib-0044] Perrow, C. (1999). Normal accidents: Living with high‐risk technologies. Princeton Univ. Press.

[bjso12805-bib-0045] Platow, M. J. , Haslam, S. A. , Reicher, S. D. , & Steffens, N. K. (2015). There is no leadership if no‐one follows: Why leadership is necessarily a group process. International Coaching Psychology Review, 10(1), 20–37.

[bjso12805-bib-0046] Reicher, S. , & Hopkins, N. (1996). Self‐category constructions in political rhetoric; an analysis of Thatcher's and Kinnock's speeches concerning the British miners' strike (1984–5). European Journal of Social Psychology, 26(3), 353–371.

[bjso12805-bib-0047] Reicher, S. , & Hopkins, N. (2000). Self and nation. Sage.

[bjso12805-bib-0600] Reicher, S. , Haslam, S. A., & Hopkins, N. (2005). Social identity and the dynamics of leadership: Leaders and followers as collaborative agents in the transformation of social reality. The Leadership Quarterly, 16(4), 547–568.

[bjso12805-bib-0048] Riggio, R. E. , & Newstead, T. (2023). Crisis leadership. Annual Review of Organizational Psychology and Organizational Behavior, 10, 201–224.

[bjso12805-bib-0049] Samad, A. , Al Jerjawi, K. , & Dadich, A. (2022). Crisis leadership: Political leadership during the COVID‐19 pandemic. Sustainability, 15(1), 266.

[bjso12805-bib-0050] Selvanathan, H. P. , Crimston, C. R. , & Jetten, J. (2022). How being rooted in the past can shape the future: The role of social identity continuity in the wish for a strong leader. The Leadership Quarterly, 33(4), 101608.

[bjso12805-bib-0051] Sriharan, A. , Hertelendy, A. J. , Banaszak‐Holl, J. , Fleig‐Palmer, M. M. , Mitchell, C. , Nigam, A. , Gutberg, J. , Rapp, D. J. , & Singer, S. J. (2022). Public health and health sector crisis leadership during pandemics: A review of the medical and business literature. Medical Care Research and Review, 79(4), 475–486. 34474606 10.1177/10775587211039201 PMC9218413

[bjso12805-bib-0052] Steffens, N. K. , Haslam, S. A. , Reicher, S. D. , Platow, M. J. , Fransen, K. , Yang, J. , Ryan, M. K. , Jetten, J. , Peters, K. , & Boen, F. (2014). Leadership as social identity management: Introducing the identity leadership inventory (ILI) to assess and validate a four‐dimensional model. The Leadership Quarterly, 25(5), 1001–1024.

[bjso12805-bib-0053] Steffens, N. K. , Munt, K. A. , van Knippenberg, D. , Platow, M. J. , & Haslam, S. A. (2021). Advancing the social identity theory of leadership: A meta‐analytic review of leader group prototypicality. Organizational Psychology Review, 11(1), 35–72.

[bjso12805-bib-0054] Tajfel, H. (1974). Social identity and intergroup behaviour. Social Science Information, 13(2), 65–93.

[bjso12805-bib-0055] Tajfel, H. , Turner, J. C. , Austin, W. G. , & Worchel, S. (1979). An integrative theory of intergroup conflict. In Organizational Identity: A Reader (pp. 33–37). Brooks/Cole.

[bjso12805-bib-0056] Templeton, A. , Guven, S. T. , Hoerst, C. , Vestergren, S. , Davidson, L. , Ballentyne, S. , Madsen, H. , & Choudhury, S. (2020). Inequalities and identity processes in crises: Recommendations for facilitating safe response to the COVID‐19 pandemic. British Journal of Social Psychology, 59(3), 674–685. 32583423 10.1111/bjso.12400 PMC7383992

[bjso12805-bib-0057] Turner, J. , Hogg, M. , Reicher, O. P. , Reicher, S. D. , & Wetherell, M. S. (1987). Rediscovering the social group: A self‐categorization theory. Blackwell.

[bjso12805-bib-0500] Uhl‐Bien, M. , & Arena, M. (2018). Leadership for organizational adaptability: A theoretical synthesis and integrative framework. The Leadership Quarterly,, 29(1), 89–104.

[bjso12805-bib-0059] Van Dick, R. , Lemoine, J. E. , Steffens, N. K. , Kerschreiter, R. , Akfirat, S. A. , Avanzi, L. , Dumont, K. , Epitropaki, O. , Fransen, K. , & Giessner, S. (2018). Identity leadership going global: Validation of the identity leadership inventory across 20 countries. Journal of Occupational and Organizational Psychology, 91(4), 697–728.

[bjso12805-bib-0060] Weick, K. E. , & Roberts, K. H. (1993). Collective mind in organizations: Heedful interrelating on flight decks. Administrative Science Quarterly, 38, 357–381.

[bjso12805-bib-0061] Williams, T. A. , Gruber, D. A. , Sutcliffe, K. M. , Shepherd, D. A. , & Zhao, E. Y. (2017). Organizational response to adversity: Fusing crisis management and resilience research streams. Academy of Management Annals, 11(2), 733–769.

[bjso12805-bib-0062] Wilson, S. , & Newstead, T. (2022). The virtues of effective crisis leadership: What managers can learn from how women heads of state led in the first wave of COVID‐19. Organizational Dynamics, 51(2), 100910. 35342206 10.1016/j.orgdyn.2022.100910 PMC8940566

[bjso12805-bib-0063] Wu, Y. L. , Shao, B. , Newman, A. , & Schwarz, G. (2021). Crisis leadership: A review and future research agenda. The Leadership Quarterly, 32(6), 101518.

